# A bibliometric analysis of the top 100 most-cited articles in interventional cardiology: trends, authors, journals, origins, and gender representation

**DOI:** 10.1097/MS9.0000000000003928

**Published:** 2025-09-23

**Authors:** Ali Abdullah, Muhammad Abdul Qadeer, Adeena Jamil, Zain Ali Nadeem, Amber Siddique, Hiba Azhar, Syeda Elezeh Sabahat, Sejal Jain Kailash, Hafeez Ul Hassan Virk

**Affiliations:** a Department of Medicine, Jinnah Sindh Medical University, Karachi, Pakistan; b Department of Medicine, Dow International Medical College, Dow University of Health Sciences, Karachi, Pakistan; c Department of Medicine, Allama Iqbal Medical College, Lahore, Pakistan; d Department of Medicine, Faisalabad Medical University, Allied and DHQ Hospitals, Faisalabad, Pakistan; e Department of Medicine, Vinnytsia National Medical University, Vinnytsia, Ukraine; f Harrington Heart & Vascular Institute, Case Western Reserve University, University Hospitals Cleveland Medical Center, Cleveland, OH, USA

**Keywords:** authorship analysis, bibliometric analysis, citation trends, gender representation in research, interventional cardiology

## Abstract

**Introduction:**

Bibliometric analysis provides a quantitative and objective method to identify research trends, influential authors, and institutions within a field, guiding both researchers and funding bodies.

**Aim:**

To identify and evaluate the top 100 most-cited articles in interventional cardiology, analyzing citation trends, authorship, funding, geographic origins, and gender representation.

**Methods:**

A systematic search was conducted on Scopus in June 2023 by two independent reviewers to identify the top 100 most-cited interventional cardiology articles. Both original and review articles were included regardless of publication year or language. Guideline articles and non-human studies were excluded. Extracted data included citation counts, publication year, study design, author affiliations, gender of first and senior authors, and funding status. Statistical analysis was performed using SPSS v25.0.

**Results:**

The 100 articles received a total of 225 984 citations, with a median of 1640.5 and 100.4 citations per year. Most were randomized controlled trials (61%) and original research (98%), primarily published between 1993 and 2012. *The New England Journal of Medicine* and *The Lancet* published the majority of these, with the USA contributing 76 articles. Among 747 authors, only 10% of first authors and 14.6% of senior authors were female. No significant correlation was found between gender or funding and citation count.

**Conclusion:**

This analysis reveals key trends in interventional cardiology, emphasizing the dominance of high-income countries and underrepresentation of women and developing regions. Greater inclusivity and global collaboration are needed to foster a more equitable research environment.

## Introduction

Bibliometric analyses have risen in popularity in the last few decades, owing to their objective and quantitative nature as tools to analyze published literature. Initially, they involved citation analysis, which helps identify the most impactful articles in a field. Over time, bibliometrics analyses have evolved to include geographical, authorship, and institutional analyses, allowing prospective researchers to better establish frameworks for future research by choosing different populations^[1]^. In recent years, it has been indicated that highly cited and innovative researchers need to receive proper funding, and the identification of such individuals is a critical aspect in alleviating such concerns^[2]^. Bibliometrics serve as yardsticks to measure the hotspots of research in different fields, specialties, and subspecialties, which allows researchers and funding agencies to properly allocate their resources and pay due attention to under-researched areas^[3]^. Bibliometric analyses have been conducted in various specialties and subspecialties^[4–7]^.

Interventional cardiology focuses on catheter-based procedures to deal with structural anomalies of the heart. It deals with nonsurgical, catheter-based procedures and imaging techniques to diagnose and treat cardiovascular diseases^[8,9]^. The most common applications of interventional cardiology include the treatment of coronary artery disease^[10,11]^, heart valve disorders^[12–15]^, and congenital heart defects^[16,17]^.

Previously, bibliometric analysis has been conducted on interventional cardiology^[18]^, but many new horizons have emerged since^[19–21]^, which warrant further exploration. We aim to present the most highly cited articles and analyze their study type, country of origin, funding, and publication trends. Additionally, we seek to determine the most impactful authors and identify gender representation in this field. This study complies with the TITAN guidelines 2025^[22]^.

## Methodology

In June 2023, two independent reviewers conducted a search for the top 100 most-cited articles related to Interventional Cardiology. The approach of using two reviewers reduces bias and was first introduced by Lim et al^[23]^. The articles were extracted from various journals using the Scopus Library database, which is available online at www.scopus.com. It is worth noting that Scopus is known for its comprehensive coverage of articles, surpassing other alternatives such as PubMed or Web of Science. It is also known for its faster citation analysis^[24]^. Only articles that primarily or majorly focused on Interventional Cardiology were included in the selection.

Previous review articles and PubMed Medical Subject Headings were also thoroughly searched in order to extract the relevant words. To accommodate spelling differences and plurals, we employed a wildcard character (*). Additionally, we included common abbreviations like PCI (percutaneous coronary intervention), PTCA (percutaneous transluminal coronary angioplasty), and FFR (fractional flow reserve). The search string used was (“coronary angiogra*” OR “coronary angioplasty” OR “percutaneous” OR “percutaneous valv*” OR “catheter” OR “stent*” OR “atherectomy” OR “transcatheter” OR “ablation” OR “coronary arteriograph*” OR “fractional flow reserve” OR ffr OR “thrombectomy” OR “pacemaker insert*” OR “defibrillator implant*” OR “percutaneous coronary intervention” OR pci OR “percutaneous transluminal coronary angioplasty” OR ptca OR “interventional cardiology” OR “cardiac intervention” OR “coronary intervention” OR “coronary stent*” OR “balloon angioplast*” OR “cardiac catheter*” OR “transcatheter aortic valve replacement” OR tavr OR “structural heart intervention” OR “interventional vascular cardiology” OR “intravascular imag*” OR “minimally invasive cardiology”). We meticulously examined the articles, abstracts, and keywords for the specified terms. The reviewers conducted the searches on Scopus during the same week without knowing each other’s findings. The initial search yielded a total of 1 007 719 documents. Our inclusion criteria encompassed both original and review articles while excluding guideline articles due to their tendency to attract a high number of citations. To ensure precision, articles were selected strictly based on their alignment with the inclusion criteria. All abstracts of the retrieved articles were thoroughly evaluated to ascertain their relevance to the topic and eligibility for inclusion. When abstracts were unavailable on Scopus, we sought them from alternative sources like PubMed and Web of Science. To ensure a comprehensive approach, the inclusion criteria encompassed studies without available abstracts, and studies written in languages other than English. Studies on non-human subjects were, however, excluded. There were no specific limitations regarding the time interval for the inclusion or exclusion of publications, and the search encompassed all journals present in the database.

**HIGHLIGHTS**

- Interventional cardiology research peaked in 2003–2012 (*n* = 46).
- Fifty-three top-cited papers appeared in *The New England Journal of Medicine*.
- The USA led globally in research output, following Canada and Europe (*n* = 76).
- Women made up only 10% of authors – highlighting a gender gap in authorship.
- Government-funded studies (25%) showed no link to citations or study outcomes.

The chosen articles were organized using the “cited by” feature. Subsequently, a final list of the top 100 most cited articles, arranged in descending order, was compiled and compared between the two reviewers. A slight discrepancy of 4.3% was observed in the lists. To resolve these differences, discussions were held until a consensus was reached. Once the final list was established, it was exported to Microsoft Excel 2021 for a comprehensive analysis.

For each article, essential information such as citation count, publication year, country of origin, number of authors, and the journal it was published in was extracted. The authors’ affiliations and their H-indexes were obtained from Scopus. Citations-per-year values were calculated for all articles. Furthermore, the impact factor (IF) of the respective journals was retrieved from Thomson Reuters Journal Citation Reports 2022.

To gain insights into the distribution of citations, the mean, median, and interquartile ranges were calculated for both the total number of citations and the number of citations per year. Additionally, in situations where authors had affiliations from multiple countries or institutes, Scopus attributed the article to multiple origins.

Determining the gender of the first and senior authors (last author) was done using genderize.io. A probability of 1.0 was taken as a confirmation for the gender. In cases where doubt remained (any probability < 1.0) about their gender, their pictures on the institutional website were examined and the pronouns used to address them were noted. For articles with a single author, that individual was considered the first author.

The data visualization elements, such as tables and charts, were created utilizing Microsoft Word and Excel, respectively. To assess statistical significance, the tests were conducted using SPSS version 25.0, developed by IBM Corp. in Armonk, NY, USA. The Spearman correlation test was utilized to assess, both, the relationship between a journal’s IF and the total number of citations it received, as well as its association with the number of articles listed. To investigate the connection between funding and gender with the number of citations, the Mann–Whitney *U* test was applied. The chi-squared (χ^2^) test was employed to determine whether there was an association between the sex of the first and senior authors. The χ^2^ test was also applied to determine if an association existed between the presence of funding and the outcome of the study being positive. In all cases, a *P*-value of less than 0.05 was considered significant. It is worth noting that ethical approval was not required for this study, as it solely involved analyzing previously published data.

## Results

### Citation count, average citations per year, and article type

Table 1 presents a compilation of the 100 most referenced articles in the field of interventional cardiology. The number of citations for these top 100 articles varied from 1124 to 5875 citations, with a median value of 1640.5 (interquartile range of 1770.3). Collectively, these articles garnered a total of 225 984 citations. The average citations per year for each article ranged from 27 to 654, with a median of 100.4 and a mean of 163.3 (interquartile range of 126.2).

**Table 1:** Top 100 articles in interventional cardiology with their total citations and average citations per year

Rank	Title	Total citations	Average citations per year
1	Quantification of coronary artery calcium using ultrafast computed tomography^[25]^	5875	178
2	Prophylactic implantation of a defibrillator in patients with myocardial infarction and reduced ejection fraction^[26]^	5762	274
3	Transcatheter aortic-valve implantation for aortic stenosis in patients who cannot undergo surgery^[27]^	5580	429
4	Amiodarone or an implantable cardioverter-defibrillator for congestive heart failure^[28]^	5447	303
5	Transcatheter versus surgical aortic-valve replacement in high-risk patients^[29]^	4912	409
6	Cardiac-resynchronization therapy with or without an implantable defibrillator in advanced chronic heart failure^[30]^	4878	257
7	Clinical end points in coronary stent trials: a case for standardized definitions^[31]^	4777	299
8	A randomized trial of intraarterial treatment for acute ischemic stroke^[32]^	4751	594
9	A comparison of balloon-expandable-stent implantation with balloon angioplasty in patients with coronary artery disease^[33]^	4399	152
10	Randomized assessment of rapid endovascular treatment of ischemic stroke^[34]^	4329	541
11	Endovascular thrombectomy after large-vessel ischaemic stroke: a meta-analysis of individual patient data from five randomised trials^[35]^	4198	600
12	A randomized comparison of coronary-stent placement and balloon angioplasty in the treatment of coronary artery disease^[36]^	4162	144
13	Sirolimus-eluting stents versus standard stents in patients with stenosis in a native coronary artery^[37]^	4093	205
14	Endovascular therapy for ischemic stroke with perfusion-imaging selection^[38]^	4073	509
15	A randomized comparison of a sirolimus-eluting stent with a standard stent for coronary revascularization^[39]^	3902	186
16	Optimal medical therapy with or without PCI for stable coronary disease^[40]^	3771	236
17	Stent-retriever thrombectomy after intravenous t-PA vs. t-PA alone in stroke^[41]^	3657	457
18	Primary angioplasty versus intravenous thrombolytic therapy for acute myocardial infarction: a quantitative review of 23 randomised trials^[42]^	3627	181
19	Improved survival with an implanted defibrillator in patients with coronary disease at high risk for ventricular arrhythmia^[43]^	3621	134
20	Thrombectomy within 8 hours after symptom onset in ischemic stroke^[44]^	3494	437
21	Transcatheter or surgical aortic-valve replacement in intermediate-risk patients^[45]^	3390	484
22	Percutaneous coronary intervention versus coronary-artery bypass grafting for severe coronary artery disease^[46]^	3367	241
23	An intervention to decrease catheter-related bloodstream infections in the ICU^[47]^	3223	190
24	Fractional flow reserve versus angiography for guiding percutaneous coronary intervention^[48]^	3138	224
25	Thrombectomy 6 to 24 hours after stroke with a mismatch between deficit and infarct^[49]^	3105	621
26	A comparison of antiarrhythmic-drug therapy with implantable defibrillators in patients resuscitated from near-fatal ventricular arrhythmias^[50]^	3053	117
27	Incidence, predictors, and outcome of thrombosis after successful implantation of drug-eluting stents^[51]^	2946	164
28	Thrombectomy for stroke at 6 to 16 hours with selection by perfusion imaging^[52]^	2714	543
29	Use of a monoclonal antibody directed against the platelet glycoprotein IIb/IIIa receptor in high-risk coronary angioplasty^[53]^	2687	93
30	A polymer-based, paclitaxel-eluting stent in patients with coronary artery disease^[54]^	2656	140
31	Percutaneous transcatheter implantation of an aortic valve prosthesis for calcific aortic stenosis: first human case description^[55]^	2619	125
32	Transcatheter aortic-valve replacement with a balloon-expandable valve in low-risk patients^[56]^	2616	654
33	Protected carotid-artery stenting versus endarterectomy in high-risk patients^[57]^	2578	136
34	Pathology of drug-eluting stents in humans: delayed healing and late thrombotic risk^[58]^	2511	148
35	Stenting versus endarterectomy for treatment of carotid-artery stenosis^[59]^	2362	182
36	Transcatheter aortic-valve replacement with a self-expanding prosthesis^[60]^	2098	233
37	Two-year outcomes after transcatheter or surgical aortic-valve replacement^[61]^	1932	176
38	A randomized comparison of antiplatelet and anticoagulant therapy after the placement of coronary-artery stents^[62]^	1931	72
39	A comparison of immediate angioplasty with thrombolytic therapy for acute myocardial infarction^[63]^	1896	63
40	Catheter replacement of the needle in percutaneous arteriography: a new technique^[64]^	1889	27
41	Surgical or transcatheter aortic-valve replacement in intermediate-risk patients^[65]^	1876	313
42	Dual-chamber pacing-or ventricular backup pacing in patients with an implantable defibrillator: The Dual Chamber and VVI Implantable Defibrillator (DAVID) Trial^[66]^	1735	83
43	Diagnostic performance of 64-multidetector row coronary computed tomographic angiography for evaluation of coronary artery stenosis in individuals without known coronary artery disease: results from the prospective multicenter ACCURACY (Assessment by Coronary Computed Tomographic Angiography of Individuals Undergoing Invasive Coronary Angiography) Trial^[67]^	1732	115
44	A new approach for catheter ablation of atrial fibrillation: mapping of the electrophysiologic substrate^[68]^	1707	90
45	A clinical trial comparing three antithrombotic-drug regimens after coronary-artery stenting^[69]^	1694	68
46	Transcatheter mitral-valve repair in patients with heart failure^[70]^	1690	338
47	Coronary artery anomalies in 126,595 patients undergoing coronary arteriography^[71]^	1686	51
48	Percutaneous closure of the left atrial appendage versus warfarin therapy for prevention of stroke in patients with atrial fibrillation: a randomised non-inferiority trial^[72]^	1680	120
49	Intraaortic balloon support for myocardial infarction with cardiogenic shock^[73]^	1669	152
50	Preventing complications of central venous catheterization^[74]^	1641	82
51	Bypass versus angioplasty in severe ischaemia of the leg (BASIL): multicentre, randomised controlled trial^[75]^	1640	91
52	Randomised placebo-controlled and balloon-angioplasty-controlled trial to assess safety of coronary stenting with use of platelet glycoprotein-IIb/IIIa blockade^[76]^	1606	64
53	Early and late coronary stent thrombosis of sirolimus-eluting and paclitaxel-eluting stents in routine clinical practice: data from a large two-institutional cohort study^[77]^	1591	99
54	Updated worldwide survey on the methods, efficacy, and safety of catheter ablation for human atrial fibrillation^[78]^	1581	122
55	Radial versus femoral access for coronary angiography and intervention in patients with acute coronary syndromes (RIVAL): a randomised, parallel group, multicentre trial^[79]^	1558	130
56	Comparison of coronary bypass surgery with angioplasty in patients with multivessel disease^[80]^	1557	58
57	Percutaneous repair or surgery for mitral regurgitation^[81]^	1555	130
58	Intravascular stents to prevent occlusion and re-stenosis after transluminal angioplasty^[82]^	1545	43
59	Catheterization of the heart in man with use of a flow-directed balloon-tipped catheter^[83]^	1526	29
60	Diagnostic performance of coronary angiography by 64-row CT^[84]^	1520	101
61	Acute renal failure after coronary intervention: incidence, risk factors, and relationship to mortality^[85]^	1487	57
62	Incidence and prognostic importance of acute renal failure after percutaneous coronary intervention^[86]^	1476	70
63	Endarterectomy versus stenting in patients with symptomatic severe carotid stenosis^[87]^	1418	83
64	Localized hypersensitivity and late coronary thrombosis secondary to a sirolimus-eluting stent: should we be cautious?^[88]^	1411	74
65	Transluminal placement of endovascular stent-grafts for the treatment of descending thoracic aortic aneurysms^[89]^	1411	49
66	Canadian implantable defibrillator study (CIDS): a randomized trial of the implantable cardioverter defibrillator against amiodarone^[90]^	1398	61
67	Diagnostic accuracy of noninvasive coronary angiography using 64-slice spiral computed tomography^[91]^	1380	77
68	Endovascular versus surgical treatment in patients with carotid stenosis in the Carotid and Vertebral Artery Transluminal Angioplasty Study (CAVATAS): a randomised trial^[92]^	1373	62
69	Intracoronary stenting without anticoagulation accomplished with intravascular ultrasound guidance^[93]^	1368	49
70	Optimal medical therapy with or without percutaneous coronary intervention to reduce ischemic burden: results from the Clinical Outcomes Utilizing Revascularization and Aggressive Drug Evaluation (COURAGE) trial nuclear substudy^[94]^	1357	90
71	Changes in collateral channel filling immediately after controlled coronary artery occlusion by an angioplasty balloon in human subjects^[95]^	1353	36
72	Late thrombosis in drug-eluting coronary stents after discontinuation of antiplatelet therapy^[96]^	1350	71
73	Stent thrombosis in randomized clinical trials of drug-eluting stents^[97]^	1344	84
74	Stenting versus aggressive medical therapy for intracranial arterial stenosis^[98]^	1340	112
75	30 day results from the SPACE trial of stent-protected angioplasty versus carotid endarterectomy in symptomatic patients: a randomised non-inferiority trial^[99]^	1336	79
76	Catheter ablation of accessory atrioventricular pathways (Wolff–Parkinson–White syndrome) by radiofrequency current^[100]^	1317	41
77	Outcomes associated with drug-eluting and bare-metal stents: a collaborative network meta-analysis^[101]^	1308	82
78	Coronary artery bypass graft surgery versus percutaneous coronary intervention in patients with three-vessel disease and left main coronary disease: 5-year follow-up of the randomised, clinical SYNTAX trial^[102]^	1293	129
79	Catheter ablation for atrial fibrillation with heart failure^[103]^	1269	254
80	Can coronary angiography predict the site of a subsequent myocardial infarction in patients with mild-to-moderate coronary artery disease?^[104]^	1266	36
81	Circumferential radiofrequency ablation of pulmonary vein ostia: a new anatomic approach for curing atrial fibrillation^[105]^	1262	55
82	Percutaneous coronary intervention of functionally nonsignificant stenosis: 5-year follow-up of the DEFER study^[106]^	1259	79
83	Angiographic progression of coronary artery disease and the development of myocardial infarction^[107,108]^	1241	35
84	Patterns and mechanisms of in-stent restenosis: a serial intravascular ultrasound study^[108]^	1239	46
85	5-year outcomes of transcatheter aortic valve replacement or surgical aortic valve replacement for high surgical risk patients with aortic stenosis (PARTNER 1): a randomised controlled trial^[109]^	1225	153
86	Angiographic assessment of myocardial reperfusion in patients treated with primary angioplasty for acute myocardial infarction: myocardial blush grade^[110]^	1215	49
87	Coronary artery disease in peripheral vascular patients: a classification of 1000 coronary angiograms and results of surgical management^[111]^	1207	31
88	Comparison of angioplasty with stenting, with or without abciximab, in acute myocardial infarction^[112]^	1206	57
89	Time delay to treatment and mortality in primary angioplasty for acute myocardial infarction: every minute of delay counts^[113]^	1203	63
90	Coronary morphologic and clinical determinants of procedural outcome with angioplasty for multivessel coronary disease: implications for patient selection^[114]^	1200	36
91	A comparison of coronary angioplasty with fibrinolytic therapy in acute myocardial infarction^[115]^	1199	60
92	A randomized, controlled trial of the use of pulmonary-artery catheters in high-risk surgical patients^[116]^	1199	60
93	A comparison of immediate coronary angioplasty with intravenous streptokinase in acute myocardial infarction^[117]^	1183	39
94	Angiographic patterns of in-stent restenosis: classification and implications for long-term outcome^[118]^	1179	49
95	Pathological correlates of late drug-eluting stent thrombosis: strut coverage as a marker of endothelialization^[119]^	1171	73
96	Transendocardial, autologous bone marrow cell transplantation for severe, chronic ischemic heart failure^[120]^	1164	58
97	Autologous bone marrow-derived stem-cell transfer in patients with ST-segment elevation myocardial infarction: double-blind, randomised controlled trial^[121]^	1159	68
98	Safety and efficacy of mechanical embolectomy in acute ischemic stroke: results of the MERCI trial^[122]^	1156	64
99	Defibrillator implantation in patients with nonischemic systolic heart failure^[123]^	1130	161
100	Quantification of obstructive and nonobstructive coronary lesions by 64-slice computed tomography: a comparative study with quantitative coronary angiography and intravascular ultrasound^[124]^	1124	62

A diverse array of methodologies were uncovered in the analysis. Among them, randomized controlled trials emerged as the most prevalent, comprising 61 studies, while meta-analyses and reviews constituted smaller portions, with 4 and 3 studies, respectively. Additionally, the analysis revealed a presence of case reports/series, totaling 3 studies. Delving further into observational studies, which numbered 29 in total, the breakdown yielded a mix of prospective (15 studies), retrospective (7 studies), and descriptive (7 studies) approaches. This information is displayed in Table 2.

**Table 2:** Study types of the top 100 studies

Type of study	Total number
Randomized controlled trials	61
Meta-analysis	4
Review	3
Case report/series	3
Observational studies (*n* = 29)	
Prospective	15
Retrospective	7
Descriptive	7

Out of the 100 articles, 98 were original research papers and 2 were review articles. The article titled “Quantification of coronary artery calcium using ultrafast computed tomography,” published in the *Journal of the American College of Cardiology* in 1990, received the highest number of citations, totaling 5875.

However, when ranked based on their average citations per year, a different article emerged as the most impactful. “Endovascular thrombectomy after large-vessel ischaemic stroke: a meta-analysis of individual patient data from five randomised trials,” published in *The Lancet*, in 2016, obtained an average of 599.7 citations per year, making it the most influential in terms of its continuous impact over time.

### Publications years and citations trends

**Figure 1.**
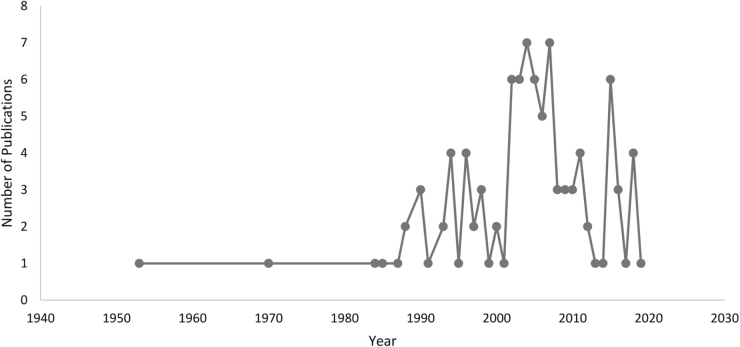
Number of articles on interventional cardiology published over the years.

**Figure 2.**
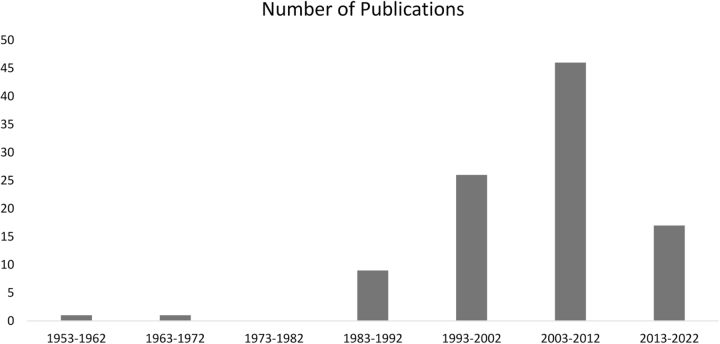
Number of articles in the top 100 list in each 10-year interval.

The top 100 articles were published between 1953 and 2019. A more thorough examination yielded significant findings and observations. The number of publications in this field displayed a gradual upward trend, as depicted in Figure 1. Initially (1953–1992), there were only one to nine publications every 10 years. However, from 1993 onward, the number of publications increased, with a notable surge occurring between 2003 and 2012, with 46 articles published, making it the most productive 10-year interval. Subsequently, the trend dipped downwards, with 17 publications from 2013 to 2022 (Fig. 2).

### Journals, institutions, and countries of origin

Table 3 displays the distribution of the top 100 articles among 11 different journals, with a notable concentration in *The New England Journal of Medicine* (*n* = 53), *Circulation* (*n* = 16), *The Lancet* (*n* = 14), and *Journal of the American College of Cardiology* (*n* = 9). The IFs of these journals, where the articles were published, ranged from 1.7 to 202.7. Our analysis revealed a statistically significant correlation between the journal’s IF and the number of top 100 cited articles (*P* < 0.05, *r* = 0.847). However, we did not find such a relationship between the IF and the total number of citations for the top 100 articles (*P* > 0.01).

**Table 3:** Journals with the highest number of articles within the top 100 list

Journal	Number of publications	Impact factor (2022)
*The New England Journal of Medicine*	53	176.08
*Circulation*	16	39.92
*The Lancet*	14	202.73
*Journal of the American College of Cardiology*	9	27.20
*Journal of the American Medical Association*	2	157.34
*Acta Radiologica*	1	1.70
*American Journal of Medicine*	1	5.93
*Annals of Surgery*	1	13.79
*Catheterization and Cardiovascular Diagnosis*	1	N/A
*Circulation: Arrhythmia and Electrophysiology*	1	7.72
*Stroke*	1	10.17

Numerous institutions were affiliated with the articles listed. The Mayo Clinic, Cleveland Clinic Foundation, and Erasmus MC emerged as the frontrunners with 12 publications each, while Cedars-Sinai Medical Center closely followed suit, contributing 10 publications. The complete list of individual institutions with 7 or more articles featured in our compilation is mentioned in Table 4.

**Table 4:** Institutes affiliated with the articles in the top 100 list

Institutes	Number of publications
Mayo Clinic	12
Cleveland Clinic Foundation	12
Erasmus MC	12
Cedars-Sinai Medical Center	10
Cardiovascular Research Foundation	8
Washington Hospital Center	8
Columbia University Irving Medical Center	8
Stanford University School of Medicine	8
University of Calgary	7
Emory University School of Medicine	7
Brigham and Women’s Hospital	7
Beth Israel Deaconess Medical Center	7
University at Buffalo, The State University of New York	7
Duke University Medical Center	7

**Figure 3.**
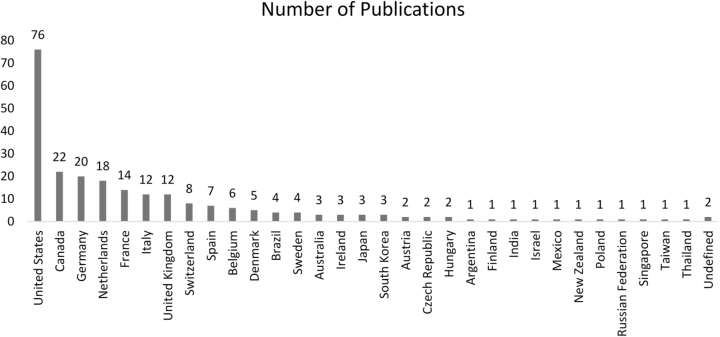
Number of articles originating from each country.

Figure 3 displays the distribution of highly cited articles among 31 different countries of origin, with the USA leading the pack (*n* = 76), contributing the majority of the articles. Other significant contributors included Canada (*n* = 27), Germany (*n* = 17), the Netherlands (*n* = 15), France (*n* = 13), Italy (*n* = 13), and the UK (*n* = 11). All other countries had fewer than 10 highly cited articles.

### Authorship

In our compilation of 100 articles, we had the contribution of 747 different authors. The number of authors per article varied, ranging from 1 to 136, and the median number of authors per article was 13. Out of these 747 authors, 159 of them had more than two articles in the list. We have presented the top authors in the field of Interventional Cardiology in Table 5, including their affiliated institutions and H-index. At the top of the list is Martin B. Leon with 13 articles, followed by Jeffrey J. Popma with 8 articles, and Antonio Colombo with 8 articles. All of the top 20 authors were male.

**Table 5:** Authors with five or more articles within the top 100 list

Author	Gender	Total publications	First position	Other positions	Last positions	Affiliation	H-Index
Leon, M.B.	Male	13	3	6	4	Columbia University Irving Medical Center, New York, United States	176
Popma, J.J.	Male	8	0	8	0	Medtronic, Inc., Minneapolis, United States	124
Colombo, A.	Male	7	1	5	1	Humanitas University, Pieve Emanuele, Italy	150
Pichard, A.D.	Male	7	0	7	0	Georgetown University, Washington, D.C., United States	102
Serruys, P.W.	Male	7	1	2	4	University of Galway, Galway, Ireland	193
Cohen, D.J.	Male	6	0	6	0	Cardiovascular Research Foundation, New York, United States	117
Stone, G.W.	Male	6	3	3	0	Icahn School of Medicine at Mount Sinai, New York, United States	164
Webb, J.G.	Male	6	0	5	1	St. Paul’s Hospital, Vancouver, Vancouver, Canada	129
Mack, M.J.	Male	6	2	4	0	Scott and White, Temple, United States	145
Anderson, W.N.	Male	5	0	5	0	Independent Researcher, Carpinteria, United States	20
Cutlip, D.E.	Male	5	1	3	1	Baim Institute for Clinical Research, Boston, United States	84
Makkar, R.R.	Male	5	0	5	0	Cedars-Sinai Medical Center, Los Angeles, United States	91
Moses, J.W.	Male	5	1	4	0	St. Francis Hospital – The Heart Center, New York, United States	104
Thourani, V.H.	Male	5	0	5	0	Emory University School of Medicine, Atlanta, United States	92

### Gender analysis of the first and last authors

The genders of the first and last authors were determined for all of the articles. Overall, female authors held 10.1% of the authorship positions. Among the first authors, females accounted for 10%, while among senior authors, females accounted for 14.6%. Our analysis did not identify any significant correlation between the gender of the first and senior authors (*P* = 0.446), nor did we find any meaningful connection between the gender of the first author and the number of citations received by their respective article (*P* = 0.907) and (*P* = 0.807).

### Sources of funding

A little over one-quarter of the studies (26) received financial support in the form of funding. It is noteworthy that 12 articles received funding from multiple sources simultaneously. Among all the funding entities, the National Heart, Lung, and Blood Institute (a US government institute) had the highest number of studies funded, 7 in total. They were followed by Boston Scientific Corporation and Medtronic, both of which are private institutes and funded 5 studies each. When accounting for the fact that many studies received funding from multiple sources, there were a total of 76 counts of funding for the 26 studies. 32.9% of these were from government sources, whereas 44.7% were from private sources. Universities/academic institutions and non-profit/charitable organizations accounted for 13.2% and 9.2% of the total sources of funding. This information has been displayed in Table 6. Upon analyzing the citation counts of funded and non-funded studies, no significant association was observed (*P* = 0.615), suggesting that the presence of funding did not notably impact the citation rates. Additionally, no association was found between the presence of funding and the outcome of the study being positive (*P* = 0.397).

**Table 6:** Sources of funding with the number of studies funded in the top 100 list

Source of funding	Studies funded
National Heart, Lung, and Blood Institute	7
Boston Scientific Corporation	5
Medtronic	5
Edwards Lifesciences	4
Abbott Laboratories	4
National Institute of Neurological Disorders and Stroke	3
AstraZeneca	2
Boehringer Ingelheim	2
Covidien	2
Deutsche Forschungsgemeinschaft	2

## Discussion

Our comprehensive analysis of the 100 most-cited articles on interventional cardiology presents a macroscopic view of current literature. These publications highlight the contemporary pivotal trends and developments in this field. Recent citation activity, distribution, and gender representation in this field can indicate concentration in key areas of landmark research, providing invaluable insights into the existing and expanding research landscape.

Most (*n* = 72) of the articles in our list were published between 1993 and 2012 demonstrating a gradual upward trend over the years, as depicted in Figure 1. Interestingly, 2003 to 2012 was the period of peak research activity within the interventional cardiology field. Analogous trends have emerged from other bibliometrics performed in the realm of cardiology^[125–128]^, fundamentally proving that research in this domain is evolving in harmony with the broader cardiology landscape. On the contrary, publications stemming from well-established specialties like general surgery and orthopedic surgery^[129,130]^, experienced their zenith in the pre-1980 era. This highlights the vigorous nature of cardiology, which requires constant amelioration and the frequent formation of up-to-date guidelines. Following this period, a decreasing slope was observed. The lack of elapsed time required for articles to gain citations and acquire significant coverage may have sparked this downward trend.

Nearly two-thirds of the most-cited articles in our top 100 list (*n* = 67) were published in the top 2 journals with respect to IF: *New England Journal of Medicine* (*n* = 53) and *The Lancet* (*n* = 14). Our findings are in accordance with Bradford’s law which suggests that most researchers acquire the majority of their citations from a handful of “core journals” in their respective area of expertise^[131,132].^ As with the previous analysis and multiple others in the field of cardiology and beyond, our analysis also revealed a statistically significant correlation between a journal’s IF and the number of most cited articles published within that journal^[18,127,128,133]^. This further underscores the notion that the standing of a journal plays a crucial role in influencing the potential impact of its articles.

Previously, coronary angioplasty and stenting have been particular areas of interest within interventional cardiology. Moreover, owing to the increasing incidence of valvular heart diseases, Khan *et al* predicted an upsurge in research focused toward transcatheter valve repair/replacement^[18]^. However, the most cited article in our list explores a trial centered around the application of ultrafast computed tomography for identifying coronary artery calcium. This outcome is hardly astonishing, considering the concurrent surge in the literature surrounding novel imaging modalities. Contemporary techniques like cardiovascular magnetic resonance have demonstrated a temporal increase in publications over the years^[128]^. However, it is to be noted that despite estimating the impact of an article in the scientific world since its publication, total citations fail to accurately illustrate its current impact. Therefore, based on average citations per year, the most impactful article in our study was a meta-analysis exploring the efficacy of endovascular thrombectomy. Meta-analyses are the pinnacle of evidence in evidence-based medicine, as they offer a precisely accurate evaluation of a clinical concern with reduced bias and have thereby become an increasingly crucial and popular investigative approach^[134]^.

Following the previously published bibliometrics done in this field, the United States upheld its position as the most prolific contributing nation. However, Canada emerged as the second-most significant contributor over the past 6 years, followed closely by European countries with minimal input from Asian (*n* = 12), South American (*n* = 6), and African (*n* = 0) nations. Similar contributions have been noted in previous bibliometric studies in cardiology and other domains^[127,135]^. Intriguingly, the marginalization of Asian and South American countries has been noteworthy in bibliometrics focused on non-communicable diseases^[130,136]^, a pattern that remains unchanged despite their alarmingly high prevalence in these regions. These persistent findings warrant greater concentration of research efforts in these regions. The partnership between nations possessing well-established research initiatives and those in the process of development can foster a global and transparent exchange within the scientific community.

Our analysis revealed that 14 individuals authored 5 or more articles within this list, signifying the presence of a selected group of dignified researchers and influential field leaders. This figure remains substantial when bibliometrics in other broader disciplines like dermatology and emergency medicine are taken into account^[133,137]^. Furthermore, the top 20 authors were all males. A study of submitted preprints and reports revealed a sharp decline in the quantity of submitted manuscripts where women held the position of first authors during the early pandemic era, particularly in medicine^[138]^. With only 10% of the authors in our list being females, interventional cardiology remains a male-dominated field. Although we did not identify a positive correlation between the gender of the first and senior authors, previous research has shown that if a senior author is a female, women are more likely to be the first authors on manuscripts likewise^[139]^.

In our analysis, around a quarter of studies (*n* = 26) received funding, with some even obtaining support from multiple sources. Noteworthy was the government-owned National Heart, Lung, and Blood Institute, which funded 7 studies. This contrasts with the earlier study, where over half of the top interventional cardiology articles received funding. However, much like their analysis, we noted no link between the presence of funding and citation counts or the presence of significant outcomes^[18]^. Considering our study encompasses the COVID-19 period, a plausible explanation might be the shift in funding agency priorities toward pandemic-related research and immediate healthcare needs, which might have redirected resources away from studies focused on non-communicable diseases. Private funding, however, continues to play a vital role.

While we strived to mitigate bias, intrinsic limitations persist in bibliometric analysis. Firstly, Scopus has been shown to possibly omit articles before the 1980s^[24,140]^. Furthermore, recently published potential landmark articles might not have appeared yet, given the time lag for citation accumulation. Thirdly, concerns have arisen regarding the effect of self-citation bias on bibliometrics, particularly when a few authors dominate high-frequency citations, as seen in our study. However, an investigation of this issue demonstrated its limited impact on bibliometrics^[141]^. Lastly, it is important to note that our analysis solely relied on Scopus as a consistent reference. Consequently, articles not indexed by Scopus could have been excluded. However, while these limitations might slightly impact the results, they are unlikely to alter the primary trends evident in this study.

### Future outlook: role of adult congenital heart disease

As survival rates among patients with congenital heart disease (CHD) continue to improve due to advances in pediatric cardiac surgery, neonatal care, and early interventional procedures, a marked increase is seen in the number of people living with CHD into adulthood^[142,143]^. This shift in demographics has created a distinct and growing population of adults with CHD (ACHD), who have different cardiovascular needs than both pediatric and general adult cardiology populations^[144]^. Despite this clinical relevance, ACHD was not prominently featured among the top 100 most-cited articles in our analysis. This is most likely due to its relatively recent emergence as a specialized subspecialty^[145]^. Its omission is an important research gap and represents a promising avenue for future scholarly focus. With the evolution of minimally invasive transcatheter therapies, such as percutaneous pulmonary valve implantation and closure of residual shunts, the role of interventional cardiology in ACHD is rapidly expanding.

In summary, our extensive analysis of the top 100 most-cited interventional cardiology articles offers a broad overview of the field’s dynamics. Our study builds on prior knowledge, highlighting evolving patterns in the origin and themes of research in this field along with their potential implications. This analysis also highlights gender disparities among authors and geographic variations in research contributions. In light of the ever-increasing burden of cardiovascular diseases, it is imperative to explore new and increased means for international collaboration within the scientific community.

## Data Availability

The data used in this study were primarily obtained from the Scopus database (www.scopus.com), which requires institutional or paid access. Due to licensing restrictions, the raw Scopus dataset cannot be publicly shared. Additionally, supplementary information, including missing abstracts, was retrieved from PubMed (www.pubmed.ncbi.nlm.nih.gov), which is publicly accessible, and Web of Science (www.webofscience.com), which also requires a subscription. Extracted summary data, including citation counts, publication years, and author affiliations, can be made available upon reasonable request from the corresponding author. The search strategy and methodology details are fully described in the manuscript to ensure reproducibility.

## Research registration unique identifying number (UIN)

No human subjects were involved in this study, hence no registration was required.
